# Experimental investigation on the characteristics of supersonic fuel spray and configurations of induced shock waves

**DOI:** 10.1038/srep39685

**Published:** 2017-01-05

**Authors:** Yong Wang, Yu-song Yu, Guo-xiu Li, Tao-ming Jia

**Affiliations:** 1 School of Mechanical, Electronic and Control Engineering, Beijing Jiaotong University, Beijing, 100044, China

## Abstract

The macro characteristics and configurations of induced shock waves of the supersonic sprays are investigated by experimental methods. Visualization study of spray shape is carried out with the high-speed camera. The macro characteristics including spray tip penetration, velocity of spray tip and spray angle are analyzed. The configurations of shock waves are investigated by Schlieren technique. For supersonic sprays, the concept of spray front angle is presented. Effects of Mach number of spray on the spray front angle are investigated. The results show that the shape of spray tip is similar to blunt body when fuel spray is at transonic region. If spray entered the supersonic region, the oblique shock waves are induced instead of normal shock wave. With the velocity of spray increasing, the spray front angle and shock wave angle are increased. The tip region of the supersonic fuel spray is commonly formed a cone. Mean droplet diameter of fuel spray is measured using Malvern’s Spraytec. Then the mean droplet diameter results are compared with three popular empirical models (Hiroyasu’s, Varde’s and Merrigton’s model). It is found that the Merrigton’s model shows a relative good correlation between models and experimental results. Finally, exponent of injection velocity in the Merrigton’s model is fitted with experimental results.

For diesel engines, the fuel spray atomization and fuel-air mixing are the key factors that affect the engine performance. It is well known that several techniques can be used to improve the fuel atomization and mixing performance, such as high fuel pressure injection^1^, high pressure compressed intake^2^, intake manifold design^3^,^4^ *et al*. High fuel pressure injection is one of the most effective methods to improve the fuel atomization^5^,^6^. However, new phenomena may occur during the fuel atomization process with increasing of injection pressure^7^,^8^. Among these phenomena, the supersonic fuel spray which break the speed of sound is an attractive phenomenon. Existing research shows that fuel jet can easily exceed the speed of sound (Mach 1) by use of modern high-pressure injection systems. It is excepted that further enhance in the injection pressure, the Mach number of fuel jet increases. Based on the interaction between spray and shock waves, the supersonic fuel atomization can be divided into two types: the active and passive case. The passive cases refer to a passive effect of supersonic flow on low speed fuel spray or droplets^9^,^10^,^11^. Generally, the passive case can occur in scramjet engines, pulse detonation engines, and shock tubes^12^,^13^,^14^,^15^,^16^. For example, the fuel spray in supersonic cross air flow in scramjet engines is the passive case discussed above^17^,^18^. While the active cases occur in the supersonic spray or droplets which generate the induced shock waves, which are induced by supersonic body^19^,^20^. It is obvious that the active cases may occur in the high/ultra-high pressure fuel spray in the DI engines^21^,^22^. However, the active cases have been scarcely studied than the passive cases. For the fuel spray atomization in vehicle engines, the effects of high/ultra-high injection pressure on the characteristics of fuel spray field are always the research hotspots^23^. There have been few research carried out on the interactions between spray and induced shock waves. But the differences between supersonic and subsonic sprays may have an significant influence on the combustion system designs, system control strategies, post-processing, etc. Therefore, knowing the mechanisms of the supersonic fuel spray will aid the development of more accurate spray models and the design of the advanced internal combustion engines. In this study, high-speed photography and Schlieren techniques are applied on the research of supersonic fuel spray atomization process, to quantitatively analyze the macroscopic characteristics of fuel spray and configurations of induced shock waves. The interaction mechanisms between shock waves and fuel spray field are also discussed. The droplet size distributions of the supersonic spray are measured using a Malvern’s Spraytec (Malvern laser particle analyzer). Consequently, comparisons between the popular Sauter Mean Diameter (SMD) models and experimental results are performed.

## Experimental Method

**Figure 1:**
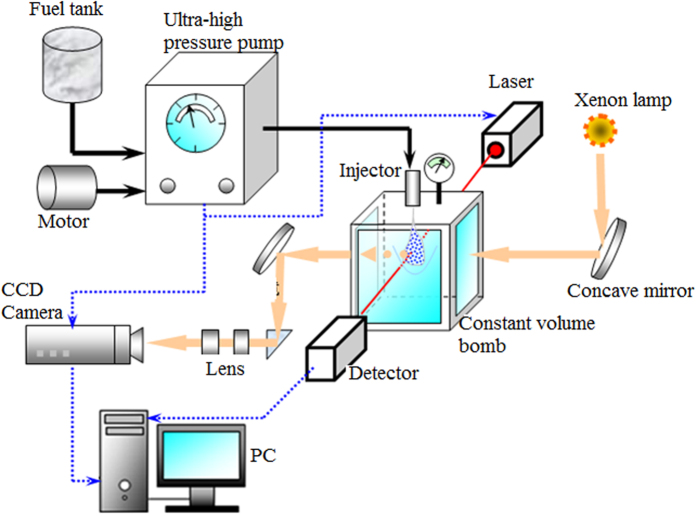
Diagram of the experimental setup.

In this study, the investigation of characteristics of supersonic fuel spray and configuration of induced shock waves is carried out with experimental method. Figure 1 shows the test platform of supersonic fuel spray designed and built by the our research group. The device consists of high pressure accumulator device, filter and fuel supply device, fuel injection control valve, high pressure oil tube, pressure gauge, motor, controller, nozzle, and fixation supports. The pressure accumulator device is designed and produced based on the principle of hydraulic to achieve the ultra-injection pressure. The operation of accumulator device is driven by the direct current (DC) motor. The switch control on the fuel injection is realized by the specially designed rapid response component. The measuring equipment of supersonic fuel spray includes a high speed camera and a Schlieren apparatus. The shock waves induced by the supersonic fuel spray can be captured by the combination of Schlieren technique and high speed camera (the high-speed photography shooting frame is set at 19,200 fps, and frame interval is 52.1 μs). The configuration of induce shock waves in the supersonic spray field is captured by Schlieren technique. Then the structural characteristics of shock waves, including the leading edge shock wave and the attached shock waves, are analyzed. A measurement of droplets diameter is achieved by the Malvern laser particle analyzer, and the position of measurement point is 30 mm away from the orifice exit along the axis of the jet. It is found that the experimental results of droplets size distribution cannot be obtained because the density spray can derail the laser through spray field if the distance between measurement point and nozzle is too short.

## Results and Discussion

In the study, the ambient pressure is 1 atm, and the ambient temperature is room temperature (the local sound velocity is around 340 m/s). To generate the supersonic fuel spray, a high enough injection pressure should be reached. The liquid injection is performed at a pressure ranging from 200 MPa to 400 MPa. The fuel spray is expected to penetrate at a maximum speed of about Mach 1.7 when the injection pressure reaches 400 MPa. The nozzle is a single-hole type, which diameter is 0.5 mm and the length of it is 3 mm. The fuel used in the test is diesel with kinematic viscosity of 5.952 × 10^−6^ m/s, surface tension coefficient of 0.0261 kg/sec^2^, and diesel density of 840 kg/m^3^.

### Supersonic fuel spray and shock wave evolution process

Figrue 2 presents a set of Schlieren photographs of the supersonic fuel spray under the injection pressure from 200 MPa to 400 MPa. The start of injection time of fuel spray is determined based on the extrapolation method of initial stage of spray penetration. It can be found that the fuel sprays under 200 MPa–400 MPa reach or exceed the local sound speed because lots of shock waves occur at the spray periphery. Under 200 MPa, the morphology of spray front is close to blunt body. There is a bow detached shock wave in front of the spray tip because the air ahead of it starts to compress. Attached shock waves occur along the spray body. According to the figures, the leading edge shock wave is wider than other shock waves due to the stronger interaction between spray tip and the air. The spray tip tends to be blunt body due to the relative weak aerodynamic effect when the injection pressure is 200 MPa. When the injection pressure is set to 300 MPa and 400 MPa, the leading oblique shock wave is formed at the initial stage of liquid injection into a gas. We can also see massive attached shock waves along the spray. Attached and detached shock waves induced by supersonic jet were experimentally observed by Nakahira, T^24^. From the figures, we can find that the intervals between attached shock waves alongside the body of the spray are almost the same even if the spray penetrates soon after injection. This implies that above phenomenon may be related to the initial flow characteristics of the fuel spray or the turbulent vortexes, which have similar coherent structure, due to gas-liquid mixing effect. The formation mechanisms of equally spaced attached shock waves remains to be further in-depth studied.

### Macroscopic characteristics of supersonic fuel spray

Figure 3 shows the influence of injection pressure on the spray tip penetration. Due to the diameter limitation of test optic windows, the upper limit of spray tip penetration in this study is 10 cm. According to the penetration characteristics, the spray penetration approximate linearly increases with time due to less effect of the air resistance relative to inertial force of the spray. With the increasing of injection pressure, the spray penetration gets longer. This result is related to both higher quantity and higher velocity of the spray.

**Figure 2:**
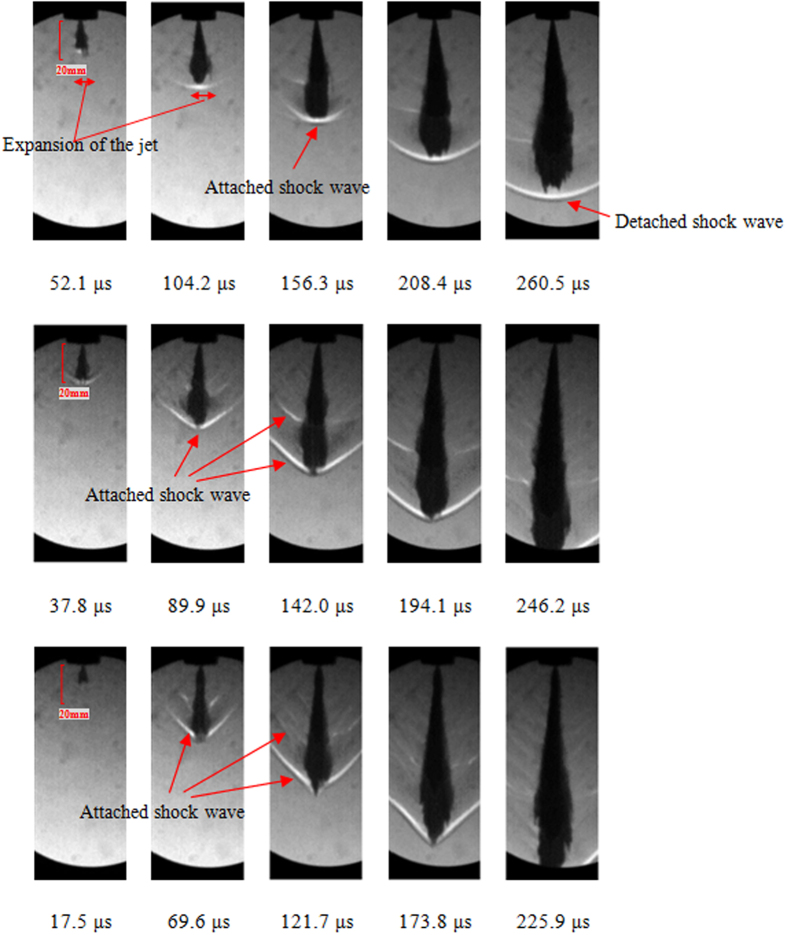
Development process of fuel spray at 200 MPa, 300 MPa, and 400 MPa.

**Figure 3:**
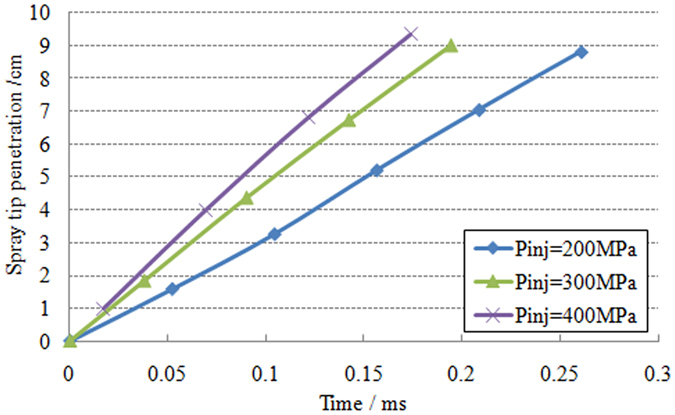
Variation of spray tip penetration over time under different injection pressure.

**Figure 4:**
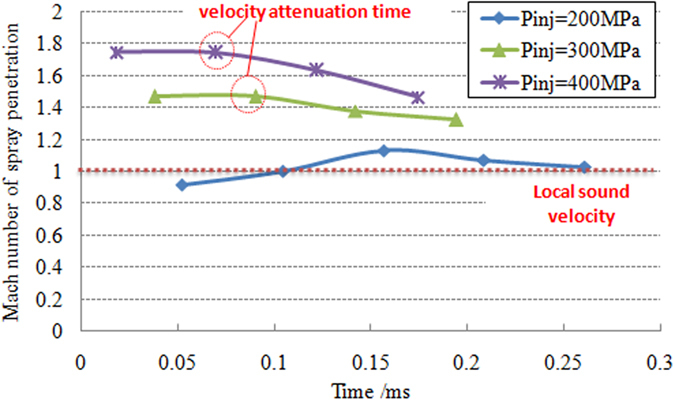
Variation of spray tip velocity over time under different injection pressure.

Figure 4 shows the temporal profiles of Mach number of the spray tip for different injection pressure cases. It can be seen that the spray tip velocity at 200 MPa is near the local sonic speed. The spray exceeds the local sound speed value after about 0.1 ms ASOI (after start of injeciton). However, a weak highlight area, which indicates density gradient change, exists in front of fuel spray at 52.1 μs after injection starting time according to the results of high-speed photography (Fig. 2). Because the measuring principle of Schlieren technique is based on the gradient of light refractive index in flow field. When injection pressure is 200 MPa, the spray tip initial velocity (t < 0.1 ms ASOI) have not reached the local sound speed (Fig. 4), which shows that the highlight leading edge wave in front of spray tip in Fig. 2 at 52.1 μs is a compressed wave rather than a shock wave. It is also known by the comparison of velocity results that the spray Mach number increases from about 1.0 (at 118 μs) to 1.17 (at 156.3 μs). When the injection pressure is set to 300 MPa and 400 MPa, the peak Mach number of spray penetration is 1.4 and 1.7 respectively during the injection. Due to the influence of the expansion of the jet and the effects of associated shock wave in the expansion process, rapid attenuation of spray tip velocity occurs after the time of peak velocity of spray tip, and the occurrence of spray tip velocity attenuation is earlier as injection pressure increases. The attenuation time is defined as the moment when the spray tip reaches its maximum velocity. In addition, the duration of spray tip velocity to maintain high speed is gradually reduced with increasing injection pressure or spray velocity. The attenuation time of spray kinetic energy for the injection pressure of 300 MPa and 400 MPa is 90 μs and 70 μs, which is obviously earlier than that of 200 MPa. The results above show that the intensity attenuation of spray kinetic energy rapidly increases with increasing spray velocity. This implies that there is a correlation between attenuation of spray kinetic energy and the wave resistance of shock wave induced by the supersonic fuel spray. When spray moves across the sonic line due to the expansion of the jet, the strong shock waves quickly deter the velocity.

**Figure 5:**
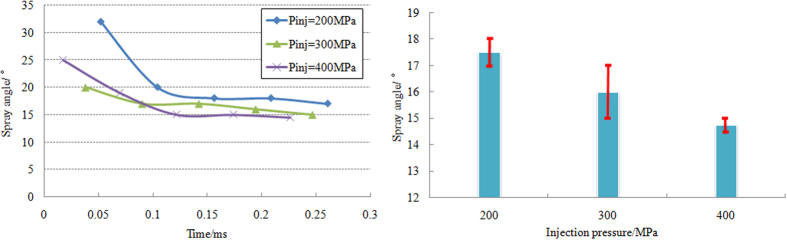
(**a**) Variation of spray cone angle over time under different injection pressure; (**b**) Influence of injection pressure on the spray cone angle in stable stage.

Figure 5(a) presents the variation of spray cone angle at injection pressure from 200 MPa to 400 MPa. A large cone angle appears at the moment of spray ejection from the nozzle orifice driven by the liquid expansion in the nozzle, instantaneous pulse of internal flow and strong aerodynamic force. And then the spray cone angle maintains stable. The experimental results show that the relatively stable spray angle (15°~18°) is achieved at around 0.1 ms after the large angle in initial period. As can be seen from Fig. 5(b), the spray cone angle in stable stage slightly reduced with increasing injection pressure.

**Figure 6:**
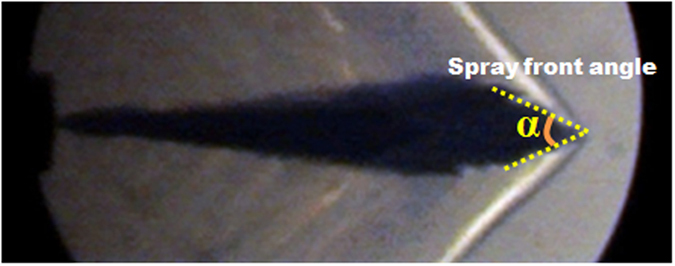
Illustration of spray front angle.

It can be concluded from the results that one of the effects of leading shock waves on the spray body is the shape change of spray tip. The existence of shock wave prevents the penetration of fuel spray, slows down the spray tip velocity and causes the shape change of the spray tip. Owing to the fact that the conventional definition of spray cone angle is determined by the upper and middle area of spray body. Obviously, the conventional definition of spray cone angle could not meet the demand of characteristic analysis on the supersonic fuel spray. Therefore, the concept of “spray front angle” is put forward aiming at the morphological characteristics of spray tip. A equivalent cone angle of spray tip, which is defined as the angle of two tangent lines to the spray tip, is used as the spray front angle as shown in Fig. 6. It is excepted that the spray front angle would be a feasible parameter that could recover the correlation or interaction between spray and the leading shock wave.

**Figure 7:**
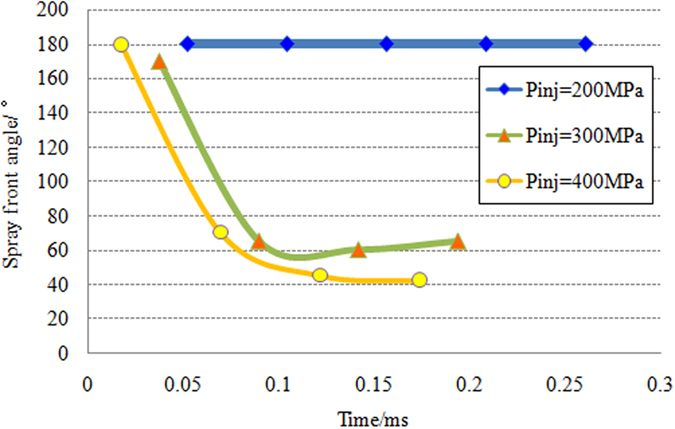
Variation of spray front angle over time under different injection pressure.

Based on the images of spray development in Fig. 2, the spray leading edge moves forward generally in a cone shape, which is consistent with the phenomenon of K. Pianthong^25^. Figure 7 shows the variation of spray front angle under different injection pressure. It is found that the spray front angle sharply decreases from 180° to 40°~70° after the injection pressure is more than 300 MPa. And then the leading edge of fuel spray moves forward in cone-shaped body with stable angle. It can also be seen that the leading edge of supersonic fuel spray tends to form a cone with the increasing spray velocity, which is conical in the Schlieren plane. According to the experimental results, when the injection pressure is high enough (up to 300 MPa and 400 MPa), there is a sudden decrease for the spray front angle after a short time ASOI. From the Fig. 2, we can find that the spray tip similar blunt body at the initial stage of injection. With the formation of strong intensity oblique shock waves, the spray tip changes into a sharp body in a short time. The reason for this phenomena may be that the strong leading edge shock wave force the spray tip, which is deformable fluid instead of solid, to deform to reach a new force balance.

### Characteristics of shock wave induced by supersonic fuel spray

**Figure 8:**
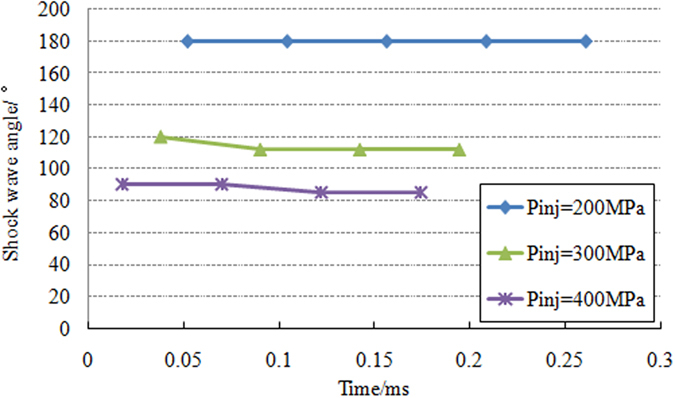
Variation of leading edge shock wave angle over time under different injection pressure.

The phenomenon of induced shock wave is the critical characteristic of supersonic fuel spray which is different from the subsonic spray. Due to the high intensity of leading edge shock wave, its structure in Schlieren images is clearer than that of attached shock waves. Additionally, the leading edge shock wave has a significant effect on the penetration behavior of fuel spray and morphology of spray tip. A quantitative analysis of the structure of induced shock waves is conducted as shown in Fig. 8. When the injection pressure is 200 MPa, a normal shock wave appears in front of spray tip. However, when the injection pressure is set to 300 MPa and 400 MPa, oblique shock waves occur at the initial stage. The shock wave angles are 112° and 85° respectively for above two injection pressure. The results show that the leading edge shock wave angles decrease with the increase of injection pressure or spray velocity.

### Distribution of droplets size in supersonic spray field

**Figure 9:**
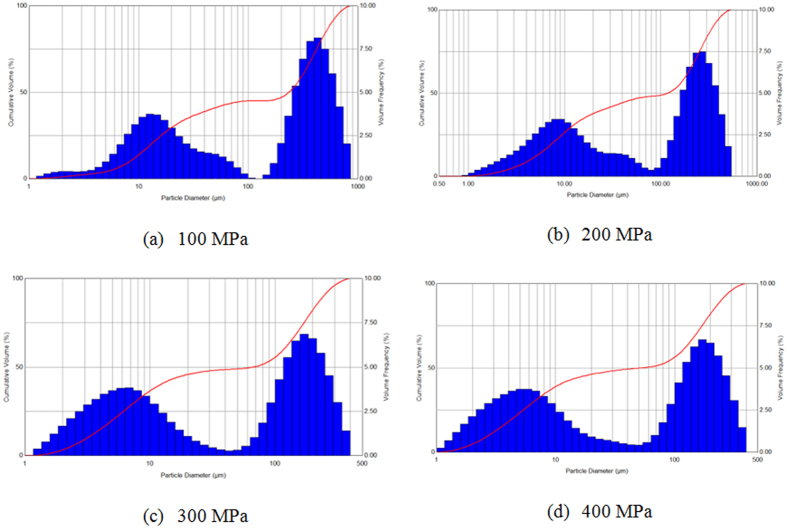
Spray droplets size distribution under different injection pressure.

It is well known that the injection pressure has a great effect on droplets breakup by increasing the relative velocity of gas-liquid phase. Figure 9 shows the results of droplet size measurement conducted in this study under the injection pressure of 100 MPa, 200 MPa, 300 MPa and 400 MPa. The figure presents the volume fraction of different droplet size which is measured by laser particle analyzer, bimodal distribution of volume fraction of droplet size appears in all four injection pressure cases.

As can be seen from Fig. 9, the first peak of the distribution locates in the large droplets size range (100 μm~500 μm), and the second one locates in the small droplets range (1 μm~20 μm). It is known that this kind of volume fraction distribution in bimodal is the typical characteristics of the high pressure fuel spray. As the injection pressure increases, the corresponding droplets size of two peaks in the volume fraction distribution gradually decreases with less amplitude. The droplet size of the right peak values at four injection pressures are 464 μm, 293 μm, 185 μm and 180 μm respectively. The droplet size of the left peak values at four injection pressures are 14 μm, 9 μm, 7 μm and 6 μm respectively. The bimodal distribution may result from a process involving breakup of large particles, multiple sources of particles and different wave growth mechanisms in the spray field.

Table 1 gives the measurement results of four average cumulative distributions under injection pressures from 100 MPa to 400 MPa. It is concluded that all these droplets size decrease but the rate of decrease reduces, which means that the effect of injection pressure on the reduction of droplets diameter has been weakening with increasing injection pressure.

It has now been found some empirical models based on the experimental data are adapted to estimate the SMD (Sauter Mean Diameter, i.e. D32) of atomized droplets in the spray field. Table 2 lists three popular empirical SMD models.

**Figure 10:**
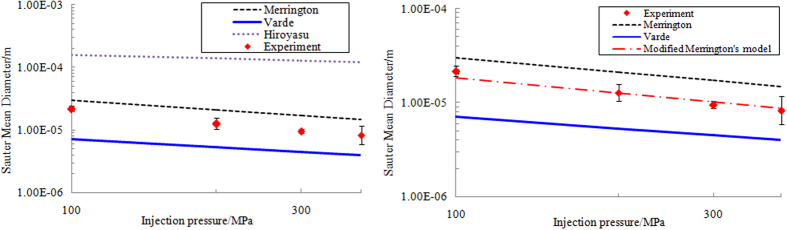
SMD results comparison of experiments and empirical models. (**a**) Comparison between experiment and current models. (**b**) Comparison between experiment and modified model.

Figure 10(a) shows the SMD results comparison of three empirical models in Table 2 with experimental results. It is found that the SMD estimated by Merrigton’s model is most similar to the experimental results. However, when the injection pressure rises, the deviation between experimental and calculated SMD predicted by Merrigton’s model increases. The worst prediction among above three models is given by Hiroyasu model. Then we redetermine the exponent (1.09) of the velocity for Merrigton’s model. Figure 10(b) presents the comparison results of modified Merrigton’s model (SMD = 500d_0_^1.2^ν_l_^0.2^/V_inj_^1.09^) with experimental data. The coefficient of determination R^2^ (R^2^ = 0.952) shows a good correlation between the modified model and experimental results.

## Conclusion

In this study, the atomization process of supersonic fuel spray is investigated under the injection pressure from 200 MPa to 400 MPa to quantitatively analyze the macroscopic characteristics of supersonic fuel spray and the structural features of shock waves. The main conclusion are as follows.

1. When the Mach number of fuel spray is locates in the transonic region of fuel spray (Ma < 1.2), the spray tip is closer to blunt body. Due to the influence of the expansion of the jet and the effects of associated shock wave in the expansion process, the type of leading edge shock wave gradually evolves from attached to detached shock wave. When the spray Mach number exceeds 1.2, the leading shock evolves from a bow shock wave to an oblique shock wave.
2. The occurrence of spray tip velocity attenuation is earlier as injection pressure increases. Additionally, the degree of kinetic energy attenuation rapidly increases with increasing Mach number. The results above show that the intensity attenuation of spray kinetic energy rapidly increases with increasing spray velocity.
3. The concept of “spray front angle” is introduced to analyze the interaction between the spray and leading edge shock wave. The results show that the spray front angle decreases with the increasing spray tip velocity, making the spray tip tend to form a cone affected and restricted by the induced oblique shock wave.
4. The leading shock wave angle decreases with the increase of spray Mach number. When injection pressure is 200 MPa, the induced leading shock is normal shock. But when injection pressure up to 300 MPa or 400 MPa, a oblique leading edge shock wave appears. The shock wave angles are 112° and 85° respectively for above two injection pressure.
5. The SMD results estimated by Merrigton’s model compared with Hiroyasu’s and Varde’s models are most similar to the experimental results for high pressure fuel spray. However, when the injection pressure rises, the deviation between predicted result of Merrigton’s model and experimental data is increased. The predicted SMD by modified Merrigton’s model (SMD = 500d_0_^1.2^ν_l_^0.2^/V_inj_^1.09^) is in good agreement with the test results.

## Additional Information

**How to cite this article**: Yong, W. *et al*. Experimental investigation on the characteristics of supersonic fuel spray and configurations of induced shock waves. *Sci. Rep.* **7**, 39685; doi: 10.1038/srep39685 (2017).

**Publisher's note:** Springer Nature remains neutral with regard to jurisdictional claims in published maps and institutional affiliations.

## Figures and Tables

**Table 1:** Results of D32, D10, D50, and D90 under injection pressure from 100 MPa to 400 MPa.

Injection pressure/MPa	D32/μm	D10/μm	D50/μm	D90/μm
100	21.4	8.3	234.5	570.5
200	12.7	4.4	118.4	349.9
300	9.3	3.1	67.1	243.5
400	8.2	2.7	52.3	244.6

**Table 2:** Empirical models for SMD of fuel spray.

Empirical models	Equation
Hiroyasu^26^	SMD = 2.33*10^−3^∆p^−0.135^ρ_g_^0.121^m_l_^0.131^
Varde^27^	SMD = 8.7d_0_(Re_l_We_l_)^−0.28^
Merrigton^28^	SMD = 500d_0_^1.2^ν_l_^0.2^/V_inj_
